# Effects of the RGD loop and C-terminus of rhodostomin on regulating integrin αIIbβ3 recognition

**DOI:** 10.1371/journal.pone.0175321

**Published:** 2017-04-11

**Authors:** Yao-Tsung Chang, Jia-Hau Shiu, Chun-Hao Huang, Yi-Chun Chen, Chiu-Yueh Chen, Yung-Sheng Chang, Woei-Jer Chuang

**Affiliations:** 1 Institute of Basic Medical Sciences and Department of Biochemistry and Molecular Biology, Tainan, Taiwan; 2 Institute of Biopharmaceutical Sciences, National Cheng Kung University College of Medicine, Tainan, Taiwan; Thomas Jefferson University, UNITED STATES

## Abstract

Rhodostomin (Rho) is a medium disintegrin containing a ^48^PRGDMP motif. We here showed that Rho proteins with P48A, M52W, and P53N mutations can selectively inhibit integrin αIIbβ3. To study the roles of the RGD loop and C-terminal region in disintegrins, we expressed Rho ^48^PRGDMP and ^48^ARGDWN mutants in *Pichia pastoris* containing ^65^P, ^65^PR, ^65^PRYH, ^65^PRNGLYG, and ^65^PRNPWNG C-terminal sequences. The effect of C-terminal region on their integrin binding affinities was αIIbβ3 > αvβ3 ≥ α5β1, and the ^48^ARGDWN-^65^PRNPWNG protein was the most selective integrin αIIbβ3 mutant. The ^48^ARGDWN-^65^PRYH, ^48^ARGDWN-^65^PRNGLYG, and ^48^ARGDWN-^65^PRNPWNG mutants had similar activities in inhibiting platelet aggregation and the binding of fibrinogen to platelet. In contrast, ^48^ARGDWN-^65^PRYH and ^48^ARGDWN-^65^PRNGLYG exhibited 2.9- and 3.0-fold decreases in inhibiting cell adhesion in comparison with that of ^48^ARGDWN-^65^PRNPWNG. Based on the results of cell adhesion, platelet aggregation and the binding of fibrinogen to platelet inhibited by ARGDWN mutants, integrin αIIbβ3 bound differently to immobilized and soluble fibrinogen. NMR structural analyses of ^48^ARGDWN-^65^PRYH, ^48^ARGDWN-^65^PRNGLYG, and ^48^ARGDWN-^65^PRNPWNG mutants demonstrated that their C-terminal regions interacted with the RGD loop. In particular, the W52 sidechain of ^48^ARGDWN interacted with H68 of ^65^PRYH, L69 of ^65^PRNGLYG, and N70 of ^65^PRNPWNG, respectively. The docking of the ^48^ARGDWN-^65^PRNPWNG mutant into integrin αIIbβ3 showed that the N70 residue formed hydrogen bonds with the αIIb D159 residue, and the W69 residue formed cation-π interaction with the β3 K125 residue. These results provide the first structural evidence that the interactions between the RGD loop and C-terminus of medium disintegrins depend on their amino acid sequences, resulting in their functional differences in the binding and selectivity of integrins.

## Introduction

RGD-containing disintegrins are potent integrin inhibitors that were found in snake venoms [1–4]. They are classified into small, medium, long, and dimeric disintegrins based on their size and the number of disulfide bonds [5]. Short disintegrins are composed of 41 to 51 residues and four disulfide bonds; medium disintegrins contain approximately 70 amino acids and six disulfide bonds; long disintegrins include a polypeptide with approximately 84 residues cross-linked by seven disulfide bonds; and homo- and hetero-dimeric disintegrins contain each subunit of approximately 67 residues with a total of ten disulfide bonds involved in the formation of four intrachain disulfides and two interchain disulfides [6]. A common structural feature of RGD-containing disintegrins is the presence of a solvent-exposed RGD tripeptide, which is crucial to the recognition of integrins [7]. The pairing of cysteine residues in disintegrins play an important role in exposing the RGD binding motif that mediates inhibition of platelet aggregation, neutrophil or endothelial cell function [1–7]. Disintegrins are therefore used to develop anti-platelets agents and anti-angiogenesis inhibitors for cancer [1–6].

Many studies have shown that the residues flanking the RGD motif and in the C-terminal region of disintegrins affect their integrins binding specificities and affinities [8–15]. For example, disintegrins with an ARGD**W** sequence exhibit a higher affinity for binding with integrin αIIbβ3, whereas disintegrins with an ARGD**N** sequence preferentially bind with integrins αvβ3 and α5β1 [10]. The amino acid sequences of RGD loop of rhodostomin (Rho) was mutated from RIPRGDMP to TAVRGDGP, resulting in a 196-fold decrease in the inhibition of integrin αIIbβ3 [12]. Replacing the N-terminal alanine with the proline of the RGD motif of elagantin (a disintegrin with an **A**RGDMP sequence) diminishes its ability to bind to integrin α5β1 [13]. The N-terminal proline residue adjacent to the RGD motif of Rho affects its function and dynamics [14]. Deletion and mutagenesis studies on echistatin have demonstrated that its C-terminal tail is important for its activity in inhibiting platelet aggregation [11, 15].

Many functional studies showed that the C-terminal tails of disintegrins act with the RGD loop to regulate integrins recognition [8, 11, 16–22]. For example, Marcinkiewicz *et al*. reported that the C-terminal region of echistatin supports integrin binding and plays a crucial role in the expression of ligand-induced binding site (LIBS) epitope and in the conformational changes of the integrins [11]. The C-terminal tail ^66^RWN residues of trimestatin are positioned close to the C-terminal side of the RGD loop and act as a secondary determinant of integrin-binding potency [18]. In particular, eristostatin requires an ARGDW motif and an intact C-terminus (NPWNG) to interact with both platelets and melanoma cells [19]. Eristostatin and bitistatin contain an ARGDWN motif with different C-terminal tails, and eristostatin exhibits a higher affinity to resting platelets [20, 23]. However, the structural basis and mechanism underlying how integrins are recognized by the C-terminus and RGD loop of disintegrins are unclear.

**Fig 1:**
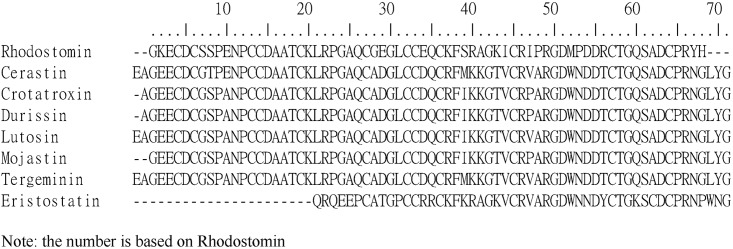
Sequence alignment of rhodostomin and disintegrins containing an ARGDWN amino acid sequence. Rhodostomin, cerastin, crotatroxin, durissin, lutosin, mojastin, tergeminin, and eristostatin are aligned, and the residues adjacent to the RGD motif and C-terminal regions are shown in boldface.

To examine how the C-terminus interacts with the RGD loop to recognize integrin αIIbβ3, we analyzed disintegrins containing an ARGDWN loop and found that they mainly exhibited C-termini with NGLYG and NPWNG amino acid sequences (Fig 1). Therefore, we used Rho as the model protein to study the effects of the ARGDWN/PRGDMP loops and C-terminal regions on the structure-activity relationships of disintegrin. Rho is obtained from *Calloselasma rhodostoma* venom and belongs to the disintegrin family [24]. It consists of 68 amino acids, including 12 residues of cysteine and a PRGDMP sequence at positions 48 to 53. We have demonstrated that Rho expressed in *Pichia pastoris* has the same function and structure as the native protein [25, 26]. In this study, we expressed Rho containing an ^48^ARGDWN or ^48^PRGDMP loop with different C-terminal sequences in *P*. *pastoris*, determined their activity in the inhibition of integrins, and used nuclear magnetic resonance (NMR) spectroscopy to compare their structural differences. We also docked these mutants into integrin αIIbβ3 and analyzed their interactions. The results demonstrated that the RGD loop and C-terminus of medium disintegrins interact with each other, resulting in structural and functional differences relevant to integrin binding.

## Materials and methods

### Expression of Rho mutants in *P*. *pastoris* and purification

The expression of Rho mutants, including ^48^PRGDMP-^65^PR, ^48^PRGDMP-^65^PRNGLYG, ^48^PRGDMP-^65^PRNPWNG, ^48^ARGDWN-^65^P, ^48^ARGDWN-^65^PR, ^48^ARGDWN-^65^PPRY, ^48^ARGDWN-^65^PRYH, ^48^ARGDWN-^65^PRNGLYG, and ^48^ARGDWN-^65^PRNPWNG, in *P*. *pastoris* and purification were accomplished by following previously described protocols [26–28].

### Mass spectrometric measurements

The molecular weights of proteins were confirmed using an LTQ Orbitrap XL mass spectrometer equipped with an electrospray ionization source (Thermo Fisher Scientific). The protein solutions (1–10 μM in 50% methanol with 0.1% formic acid) were infused into the mass spectrometer by using a syringe pump at a flow rate of 3 μL/min to acquire full scan mass spectra. The electrospray voltage at the spraying needle was optimized at 4000 V. The molecular weights of proteins were calculated by computer software Xcalibur that was provided by the Thermo Fisher Scientific.

### Cell adhesion assay

The adhesions of CHO-αIIbβ3 cells to fibrinogen, CHO-αvβ3 cells to fibrinogen, and K562 cells to fibronectin were used to determine the inhibitory activities of Rho mutants to integrins αIIbβ3, αvβ3, and α5β1. They were conducted according to previously described protocols [14, 27].

### Preparation of human platelets

Platelets were collected using 0.15 vol/vol acid-citrate dextrose (ACD) containing 85 mM trisodium citrate, 2% dextrose and 65 mM citric acid as the anticoagulant and washed using a modification of a previously described method [29]. 12 mL of blood was centrifuged at 150 × g for 10 min at room temperature (RT). The buffy coat and the red blood layers were discarded to avoid the contaminants. The remaining 5 ml of platelet-rich plasma (PRP) layer was acidified to pH 6.5 with 5 ml of ACD and then added 1 μL of 10 mM prostaglandin E1 (PGE1). Platelets were pelleted by centrifugation at 750 g for 10 min at room temperature (RT), and the supernatant was removed. The platelet pellet was gently re-suspended in 5 mL of 130 mM NaCl, 3 mM KCl, 10 mM trisodium citrate, 9 mM NaHCO_3_, 6 mM dextrose, 0.9 mM MgCl_2_, 0.81 mM KH_2_PO_4_, and 10 mM Tris (JNL) buffer at pH 7.4. Platelets were counted using a XT-1800-Hematology-Analyzer and were adjusted to 1×10^8^ per ml. Platelets were allowed to stand at RT for 45 min to let PGE1 dissipate. 20 μL of 18 mM calcium chloride was immediately added into 2 mL of platelet solution before the fibrinogen binding experiment.

### Fibrinogen binding assay

The fibrinogen (Fg) binding assay was accomplished using a modification of a previously described method [29]. Rho and its mutants (40–2000 nM), which were used as inhibitors, were added to 5 μL of 2.5 mg/mL Oregon Green 488-labeled fibrinogen (Invitrogen, UK). 20 μL aliquots of washed platelet suspension were then added and incubated for 30 min before the addition of 10 μM ADP. The resulting platelet solutions were incubated at RT for a further 30 min. The reaction was stopped by addition of 1 mL ice-cold buffer. The binding of Fg to platelets was detected using a flow cytometry. Data acquisition and analysis were performed with the Cell Quest program. Platelet populations were gated for the analysis, and the histograms of mean fluorescence were generated for each sample. Statistical analysis was performed on the geometric scale. All experiments were run in duplicate, and the reported IC_50_ values are the average of at least three separate experiments.

### Platelet aggregation assay

The inhibition of platelet aggregation by Rho mutants was accomplished by following previously described protocols [14, 27].

### Structure determination by nuclear magnetic resonance spectroscopy

Structure determination of Rho ^48^ARGDWN-^65^PRYH, ^48^ARGDWN-^65^PRNGLYG, and ^48^ARGDWN-^65^PRNPWNG mutants by NMR spectroscopy was described in detail previously [30–32]. NMR experiments were performed at 27°C on a Bruker Avance 600 spectrometer equipped with pulse field gradients and xyz-gradient triple-resonance probes. Structures were calculated using the X-PLOR program and the hybrid distance geometry-dynamical simulated annealing method [30, 31]. The structure figures were prepared using the MOLMOL [32] and PyMOL (http://www.pymol.org) programs.

### Molecular docking

The docking of Rho mutants to integrin αIIbβ3 was performed on the HADDOCK webserver by using hydrogen bond and distance restraints as described previously [33]. The starting structures for the docking were NMR structures of Rho mutants and integrin αIIbβ3 (PDB code 3ZE2) [34]. The interaction restraints were derived from the X-ray structure of integrin αIIbβ3 in complex with a GRGDSP peptide by using CCP4i software (http://structure.usc.edu/ccp4/). The selected structure cluster for the analysis was based on the lowest Z-score without any restraint violations. Hydrogen bonds and salt bridges were analyzed using PISA software (http://www.ebi.ac.uk/msd-srv/prot_int/). Cation-π interactions and non-bonded contacts were determined using CaPTURE (http://capture.caltech.edu/) and CCP4i, respectively [35].

### Protein data bank accession number and nuclear magnetic resonance assignment

The coordinates of 20 calculated structures of Rho ^48^ARGDWN-^65^PRYH, ^48^ARGDWN-^65^PRNGLYG, and ^48^ARGDWN-^65^PRNPWNG mutants were deposited in the Protein Data Bank under accession numbers 2M75, 2M7F, and 2M7H, respectively. ^1^H and ^15^N resonances of Rho ^48^ARGDWN-^65^PRYH, ^48^ARGDWN-^65^PRNGLYG, and ^48^ARGDWN-^65^PRNPWNG mutants were deposited in the BioMagResBank databank under accession numbers 19210, 19211, and 19212, respectively.

## Results

### Protein expression and purification of rhodostomin mutants

Rho mutants were expressed in *P*. *pastoris* X33 strain by using the pPICZαA vector. Recombinant Rho mutants proteins were purified to homogeneity by Ni^2+^-chelating chromatography and C18 reversed-phase HPLC. According to SDS-polyacrylamide gel electrophoresis analysis (data not shown), the purified Rho mutants proteins were homogenous. The final yields of unlabelled Rho mutants produced in *P*. *pastoris* were 10 to 25 mg/L, and the final yields of ^15^N-labeled Rho mutants were 5 to 15 mg/L.

Mass spectrometry was used to determine the molecular weights of recombinant Rho mutants. Mass spectrometry indicated that the experimental molecular weights deviated less than 1 Da from the theoretical values, which were calculated by assuming that all cysteines formed disulfide bonds in Rho mutants. For example, the experimental molecular weight of Rho ^48^ARGDWN-^65^PRYH mutant was 8464.0 Da, which was in excellent agreement with the calculated value of 8463.4 Da (Figure A in S1 File). The molecular weight of the recombinant Rho mutant had an additional 1117.2 Da from the eight extra amino acid residues (EFHHHHHH) at the N-terminus. The mass of 8463.4 Da was calculated by assuming that all cysteines formed disulfide bonds, indicating that six disulfide bonds formed in the ^48^ARGDWN-^65^PRYH mutant. The results indicated the formation of six disulfide bonds in all Rho mutants (Figure A and Table A in S1 File).

### Inhibition of integrins αIIbβ3, αvβ3, and α5β1

Inhibition of cell-expressing integrins αIIbβ3, αvβ3, and α5β1 to their ligands by Rho mutants was used to determine their activity and selectivity (Tables 1, 2 and 3). Rho and its ^48^ARGDWN- ^65^PRYH mutant inhibited the adhesion of CHO cells that expressed integrin αIIbβ3 to immobilized fibrinogen with IC_50_ values of 52.2 ± 8.2 and 162.8 ± 7.2 nM, respectively (Table 1). In contrast, Rho and its ^48^ARGDWN-^65^PRYH mutant inhibited the adhesion of CHO cells that expressed integrin αvβ3 to immobilized fibrinogen with IC_50_ values of 13.0 ± 5.7 and 246.6 ± 66.8 nM, respectively. Rho and its ^48^ARGDWN-^65^PRYH mutant inhibited integrin α5β1 adhesion to immobilized fibronectin with IC_50_ values of 256.8 ± 87.5 and 8732.2 ± 481.8 nM, respectively. Their differences in inhibiting integrins αIIbβ3, αvβ3, and α5β1 were 3.1-, 19.0-, and 34.0-fold. These results indicated that Rho containing a ^48^ARGDWN sequence exhibited selectivity for binding with integrin αIIbβ3.

**Table 1:** Inhibition of integrins αIIbβ3, αvβ3, and α5β1 by Rho and its ^48^ARGDWN mutants.

Proteins		αIIbβ3 / Fg		αVβ3 / Fg		α5β1 / Fn	
RGD Loop	C-terminus	IC_50_(nM)	*Q*	IC_50_(nM)	*Q*	IC_50_(nM)	*Q*
^48^PRGDMP	^65^PRYH	52.2±8.2	1.0	13.0±5.7	1.0	256.8±87.5	1.0
^48^ARGDWN	^65^PRYH	162.8±10.9	3.1	246.6±66.8	19.0	8732.3±481.8	34.0
Folds			3.1		19.0		34.0

*Q ratio* = IC_50_ [Rho or its mutants] / IC_50_ [Rho]

**Table 2:** Inhibition of platelet aggregation integrins αIIbβ3, αvβ3, and α5β1 by Rho ^48^ARGDWN mutants.

Proteins		αIIbβ3 / Fg		αVβ3 / Fg		α5β1 / Fn	
RGD Loop	C-terminus	IC_50_(nM)	*Q*	IC_50_(nM)	*Q*	IC_50_(nM)	*Q*
^**48**^**ARGDWN**	^**65**^**PRNPWNG**	**57.0±12.5**	**1.0**	**1207.0±73.5**	**1.0**	**2548.6±313.8**	**1.0**
^48^ARGDWN	^65^P	1314.0±121.5	23.1	868.9±87.5	0.7	7616.3±913.9	3.0
^48^ARGDWN	^65^PR	723.0±163.7	12.7	467.3±113.4	0.4	3397.0±426.1	1.3
^48^ARGDWN	^65^PRY	104.2±10.1	1.8	222.5±10.7	0.2	8938.0±1099.3	3.5
^48^ARGDWN	^65^PRYH	162.8±10.9	2.9	246.6±66.8	0.2	8732.3±481.8	3.4
^48^ARGDWN	^65^PRNGLYG	170.6±24.7	3.0	1191.8±378.7	1.0	9529.3±1224.8	3.7
Folds			1.0–23.1		0.2–1.0		1.0–3.7

*Q ratio* = IC_50_ [Rho ^48^ARGDWN mutants] / IC_50_ [Rho ^48^ARGDWN-^65^PRNPWNG mutant]

**Table 3:** Inhibition of integrins αIIbβ3, αvβ3, and α5β1 by Rho and its ^48^PRGDMP mutants.

Proteins		αIIbβ3		αvβ3		α5β1	
RGD Loop	C-terminus	IC_50_(nM)	*Q*	IC_50_(nM)	*Q*	IC_50_(nM)	*Q*
^**48**^**PRGDMP**	^**65**^**PRYH**	**52.2±8.2**	**1.0**	**13.0±5.7**	**1.0**	**256.8±87.5**	**1.0**
^48^PRGDMP	^65^PR	592.5±45.7	11.4	23.0±9.9	1.8	580.2±241.0	2.3
^48^PRGDMP	^65^PRNGLYG	186.0±11.1	3.6	26.7±3.3	2.1	238.1±19.7	0.9
^48^PRGDMP	^65^PRNPWNG	235.2±24.1	4.5	40.7±10.1	3.1	260.0±17.3	1.0
Folds			1.0–11.4		1.0–1.8		0.9–2.3

*Q ratio* = IC_50_ [Rho ^48^PRGDMP-^65^PR mutant] / IC_50_ [Rho]

We expressed a series of Rho C-terminal mutants to confirm their effects on inhibiting integrins (Table 2). Rho ^48^ARGDWN-^65^P, -^65^PR, -^65^PRY, -^65^PRYH, -^65^PRNGLYG, and -^65^PRNPWNG mutants inhibited the adhesion of CHO cells that expressed integrin αIIbβ3 to immobilized fibrinogen with IC_50_ values of 1314.0, 723.0, 104.2, 162.8, 170.6, and 57.0 nM, respectively. The ^48^ARGDWN-^65^PR mutant was 6.9-fold less active than the ^48^ARGDWN-^65^PRY mutant, suggesting that the Y67 residue may play important role in inhibiting the adhesion of integrin αIIbβ3 to immobilized fibrinogen. These Rho mutants inhibited the adhesion of CHO cells that expressed integrin αvβ3 to immobilized fibrinogen with IC_50_ values of 868.9, 467.3, 222.5, 246.6, 1191.8, and 1207.0 nM, respectively. They inhibited K562 cell adhesion to immobilized fibronectin with IC_50_ values of 7616.3, 3397.0, 8938.0, 8732.3, 9529.3, and 2548.6 nM, respectively. The affinity differences in inhibiting integrins αIIbβ3, αvβ3, and α5β1 were ranged from 1.0 to 23.1-, 0.2 to 1.0-, and 1.0 to 3.7-folds. These results demonstrated that the effect of C-terminal regions on the change of the relative binding affinity to integrins was αIIbβ3 > **αvβ3** **≥** **α5β1**. The ^48^ARGDWN-^65^PRNPWNG protein was the most selective integrin αIIbβ3 mutant and inhibited integrins αIIbβ3, αvβ3, and α5β1 with IC_50_ values of 57.0, 1207.0, and 2548.6 nM, respectively.

We also expressed Rho mutants containing a ^48^PRGDMP sequence with different C-terminal tails, including ^48^PRGDMP-^65^PR, ^48^PRGDMP-^65^PRYH, ^48^PRGDMP-^65^PRNGLYG, and ^48^PRGDMP-^65^PRNPWNG mutants, to examine the roles of the C-terminal regions (Table 3). Their affinity differences in inhibiting integrins αIIbβ3, αvβ3, and α5β1 were ranged from 1.0 to 11.4-, 1.0 to 1.8-, and 0.9 to 2.3-folds. These results indicated that the effects of C-terminal regions on the change of their relative binding affinity to integrins was αIIbβ3 > **α5β1** **≥** **αvβ3.** In contrast, the ^48^PRGDMP- ^65^PRNPWNG mutant did not exhibit any integrin selectivity and inhibited integrins αIIbβ3, αvβ3, and α5β1 with IC_50_ values of 235.2, 40.7, and 260 nM, respectively. These findings revealed that the ^48^ARGDWN sequence selectively inhibited integrin αIIbβ3. We also found that the incorporation of C-terminal NPWNG sequence with ARGDWN loop increased the inhibitory activity against integrin αIIbβ3.

### Inhibition of platelet aggregation

Recombinant Rho inhibited platelet aggregation with a K_i_ of 83.2 ± 10.4 nM, and the mutation of P48A, M52W, and P53N (^48^ARGDWN-^65^PRYH mutant) on Rho caused a 1.5-fold decrease in activity in the inhibition of platelet aggregation with a K_i_ of 128.0 ± 14.2 nM (Table 4). To study the effects of the C-terminus on the inhibition of platelet aggregation, we expressed ^48^ARGDWN-^65^P, -^65^PR, -^65^PRYH, -^65^PRNGLYG, and -^65^PRNPWNG mutants. The average IC_50_ values of ^48^ARGDWN-^65^PRNGLYG and ^48^ARGDWN-^65^PRNPWNG mutants were105.2 and 107.9 nM, respectively, which were similar to the average IC_50_ values of wild-type Rho [24]. In contrast, the average IC_50_ values of ^48^ARGDWN-^65^PR and ^48^ARGDWN-^65^PRY mutants were 235.1 and 171.4 nM, suggesting the importance of the R66 residue (Table 4). These results showed that the length of the C-terminus and the R66 residue of Rho with an ^48^ARGDWN loop sequence are essential for interacting with platelets integrin αIIbβ3.

**Table 4:** Inhibition of platelet aggregation and the binding of fibrinogen to platelets by Rho ARGDWN mutants.

Proteins		Platelet aggregation		Fibrinogen-platelet binding	
RGD Loop	C-terminus	IC_50_(nM)	*Q*^a^	IC_50_(nM)	*Q*^a^
^48^PRGDMP	^65^PRYH	83.2±10.4		90.8±28.9	
^**48**^**ARGDWN**	^**65**^**PRNPWNG**	107.9±16.1	**1.0**	141.9±33.9	**1.0**
^48^ARGDWN	^65^P	235.1±30.5	2.2	450.8±112.9	3.2
^48^ARGDWN	^65^PR	171.4±4.2	1.6	ND^b^	ND^b^
^48^ARGDWN	^65^PRY	155.1±6.7	1.4	ND^b^	ND^b^
^48^ARGDWN	^65^PRYH	128.0±14.2	1.2	187.8±67.8	1.3
^48^ARGDWN	^65^PRNGLYG	105.2±13.3	1.0	123.1±22.7	0.9
Folds			1.0–2.2		0.9–3.2

^a^*Q ratio* = IC_50_ [Rho ^48^ARGDWN mutants] / IC_50_ [Rho ^48^ARGDWN-^65^PRNPWNG mutant]

^b^ND, not determined.

We also expressed Rho mutants containing a ^48^PRGDMP sequence with different C-terminal tails to examine the C-terminal effect on inhibiting platelet aggregation (Table B in S1 File). The IC_50_ values of ^48^PRGDMP-^65^PR, ^48^PRGDMP-^65^PRYH, ^48^PRGDMP-^65^PRNGLYG, and ^48^PRGDMP- ^65^PRNPWNG proteins were 155.2, 83.2, 96.9, and 130.9 nM, respectively. These results also showed that the length and amino acid contents of the C-terminus in Rho with a ^48^PRGDMP loop sequence may play a critical role in interacting with platelets integrin αIIbβ3.

### Inhibition of the binding of fibrinogen to platelets

The activity of ARGDWN mutants to inhibit the interaction between soluble fibrinogen and washed human platelets was evaluated. The analysis showed that the IC_50_ values of ^48^ARGDWN-^65^P, ^48^ARGDWN-^65^PRYH, ^48^ARGDWN-^65^PRNGLYG, and ^48^ARGDWN- ^65^PRNPWNG proteins were 450.8, 187.8, 123.1, and 141.9 nM, respectively. In particular, ^48^ARGDWN-^65^P mutant exhibited 3.2-fold decrease in inhibiting the association between washed platelet and soluble fibrinogen in comparison with that of ^48^ARGDWN- ^65^PRNPWNG mutant. These results were consistent with the results of platelet aggregation that the length of the C-terminus and the R66 residue of ^48^ARGDWN mutants are essential for interacting with platelets integrin αIIbβ3. In contrast to the result of the adhesion of cell-expressing integrin αIIbβ3 to immobilized fibrinogen, ^48^ARGDWN-^65^P mutant exhibited significant effect with 23.1-fold decrease in activity.

### Structure determination

**Fig 2:**
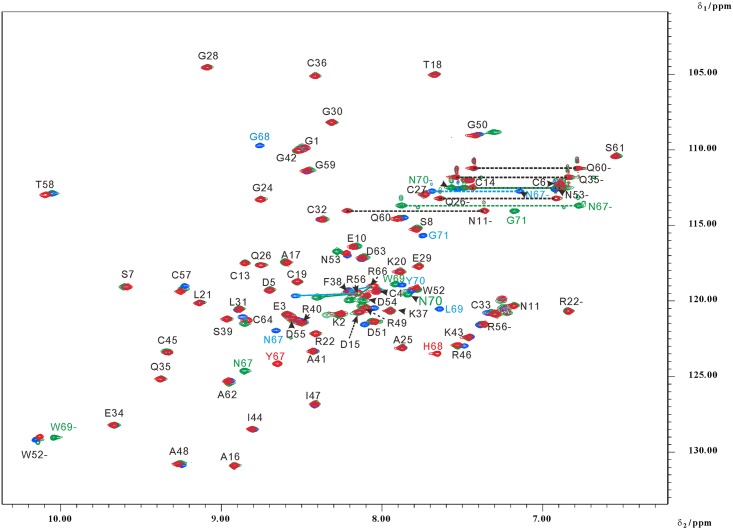
2D ^1^H-^15^N HSQC spectra of Rho ^48^ARGDWN mutants at pH 6. The peaks of Rho ^48^ARGDWN-^65^PRYH, ^48^ARGDWN-^65^PRNGLYG, and ^48^ARGDWN-^65^PRNPWNG mutants are shown in red, blue, and green. Correlation peaks are labeled according to residues type and sequence number. The peaks connected by dotted lines correspond to Gln and Asn side chain NH_2_ group. In addition, the resonances of the side chains were labeled with the—sign. The chemical shift differences larger than 0.1 ppm are connected with the arrow, and we use the spectrum of ^48^ARGDWN-^65^PRYH mutant as the reference. The blue and green arrows represented the Rho ^48^ARGDWN-^65^PRNGLYG and ^48^ARGDWN-^65^PRNPWNG mutants.

The solution structures of Rho ^48^ARGDWN-^65^PRYH, -^65^PRNGLYG, and -^65^PRNPWNG mutants expressed in *P*. *pastoris* were determined using NMR spectroscopy and the hybrid distance geometry-dynamical simulated annealing method. NOE-derived distance restraints were obtained from 2D NOESY and TOCSY and 3D ^15^N-edited TOCSY and ^15^N-edited NOESY. NMR spectra were recorded at pH 6. NMR assignments of Rho ^48^ARGDWN-^65^PRYH, -^65^PRNGLYG, and -^65^PRNPWNG mutants were obtained by analyzing standard 2D homonuclear and 3D heteronuclear NMR data (data not shown). The superimposition of the ^1^H-^15^N HSQC spectra of Rho ^48^ARGDWN-^65^PRYH, -^65^PRNGLYG, and -^65^PRNPWNG mutants showed that they exhibited the same secondary structures and tertiary fold (Fig 2). The secondary structures were identified based on networks of sequential and medium-range NOEs and ^3^J_HNα_ coupling constants (Figure B in S1 File). Their six cysteine pairs (C4–C19, C6–C14, C13–C36, C27–C33, C32–C57, and C45–C64) and three short regions of two-stranded antiparallel β-sheets (residues 13–14 and 20–21, 33–34 and 37–38, and 43–45 and 55–57) were also identified (data not shown).

According to the NOE spectra, the conformational differences were found in the C-terminal regions and their interactions with the ARGDWN loop (Figure C in S1 File). The NPWN residues of the ^48^ARGDWN-^65^PRNPWNG mutant formed a β-turn structure, which was reflected by the NOEs between H_α_ of N67 and H_N_ of N70 and between H_α_ of P68 and H_N_ of N70. In contrast, no turn structure was identified from the C-terminal regions of ^48^ARGDWN-^65^PRYH and ^48^ARGDWN-^65^PRNGLYG mutants.

The NOEs were found between the ARGDWN loop and their C-terminal regions, indicating that they were close to each other. For example, the NOEs between H_β_ of W52 and H_δ2_ and H_ε1_ of H68, between and H_δ1_ of W52 and H_β_ and H_α_ of H68, and between H_ζ2_ of W52 and H_β_ and H_α_ of H68 were found in the Rho ^48^ARGDWN-^65^PRYH mutant. The NOEs between H_δ1_ of W52 and H_δ2_ and H_δ1_ of L69, between H_ζ2_ of W52 and H_δ2_ and H_δ1_ of L69, and between H_ε3_ of W52 and H_δ2_ and H_δ1_ of L69 were found in the ^48^ARGDWN-^65^PRNGLYG mutant. The NOEs between H_Nε1_ of W52 and H_δ22_ and H_δ21_ of N70, between H_β_ of A48 and H_δ22_ and H_δ21_ of N70, and between H_α_ of A48 and H_δ22_ and H_δ21_ of N70 were found in the ^48^ARGDWN-^65^PRNPWNG mutant. The results showed that the interactions between the ARGDWN loop and C-terminal regions depend on their amino acid sequences.

**Fig 3:**
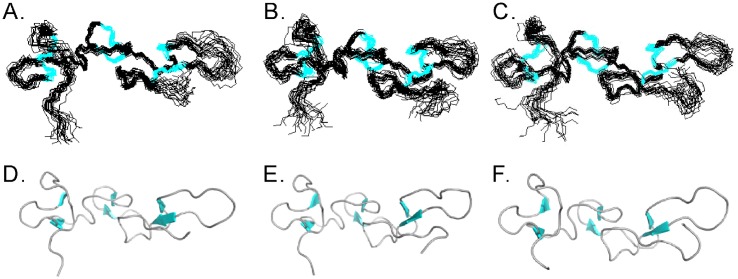
3D structures of Rho ^48^ARGDWN-^65^PRYH, ^48^ARGDWN-^65^PRNGLYG, and ^48^ARGDWN-^65^PRNPWNG mutants. Ribbon representation of 20 lowest-energy NMR structures of Rho ^48^ARGDWN-^65^PRYH **(A)**, ^48^ARGDWN-^65^PRNGLYG **(B)**, and ^48^ARGDWN-^65^PRNPWNG **(C)**. Cartoon representation of the averaged structure of Rho ^48^ARGDWN-^65^PRYH **(D)**, ^48^ARGDWN-^65^PRNGLYG **(E)** and ^48^ARGDWN-^65^PRNPWNG **(F)** mutant. The β-strands are shown in cyan. The structures are superposed on the main-chain atoms of the β-strands.

The 3D structures of the Rho ^48^ARGDWN-^65^PRYH, -^65^PRNGLYG, and -^65^PRNPWNG mutants were calculated using 1084, 1121, and 1142 experimentally derived restraints with an average of 15.9, 15.8, and 16.1 restraints per residue, respectively (Table 5). The backbone RMSD values of the Rho ^48^ARGDWN-^65^PRYH, -^65^PRNGLYG, and -^65^PRNPWNG mutants were 0.92 ± 0.16 Å, 1.05 ± 0.21 Å, and 1.09 ± 0.31 Å. The backbone RMSD values of the Rho ^48^ARGDWN-^65^PRYH, -^65^PRNGLYG, and -^65^PRNPWNG mutants for the three β-sheet regions (13–14, 20–21, 33–34, 37–38, 43–45, and 55–57) were 0.41 ± 0.11 Å, 0.48 ± 0.14 Å, and 0.45 ± 0.13 Å, respectively. Based on the Ramachandran plot analysis, all of the dihedral angles were in the acceptable region. A summary of the restraints and structural statistics is shown in Table 5. The 20 most favorable structures of mutants from 200 initial structures are shown in Fig 3.

**Table 5:** Summary of structural restraints and statistics for Rho ^48^ARGDWN mutants.

Summary of restraints and statistics	^48^ARGDWN-C-terminal mutants		
	-^65^PRYH	-^65^PRNGLYG	-^65^PRNPWNG
**Distance and dihedral angle restraints**			
Intra-residue	152	160	165
Sequential	114	125	118
Medium range	365	369	392
Long range	383	392	393
Hydrogen bond	9	9	9
Dihedral angles	55	60	59
Disulfide	6	6	6
Total	1084	1121	1142
**Energy statistics X-plor energy (kcal mol**^**-1**^**)**			
Enoe	14.96±2.87	20.51±5.72	21.30±2.96
Evdw	9.64±2.52	12.59±2.99	13.70±5.33
**Geometric statistics**			
**Deviations from idealized geometry**			
All backbone atoms (Å)	0.92±0.16	1.05±0.21	1.09±0.31
Backbone atoms (13–14, 20–21, 33–34, 37–38, 43–45, 55–57) (Å)	0.41±0.11	0.48±0.14	0.45±0.13
All heavy atoms (Å)	1.42±0.14	1.50±0.18	1.53±0.31
Heavy atoms (13–14, 20–21, 33–34, 37–38, 43–45, 55–57) (Å)	0.86±0.11	0.93±0.16	0.95±0.14
**Ramachandran analysis**			
Most favored region (%)	75.8	75.2	75.2
Additionally allowed regions (%)	21.2	20.7	20.4
Generously allowed regions (%)	2.9	4.1	4.4
Disallowed regions (%)	0.0	0.0	0.0

### Structural differences among rhodostomin ^48^ARGDWN-^65^PRYH, ^48^ARGDWN- ^65^PRNGLYG, and ^48^ARGDWN-^65^PRNPWNG mutants

**Fig 4:**
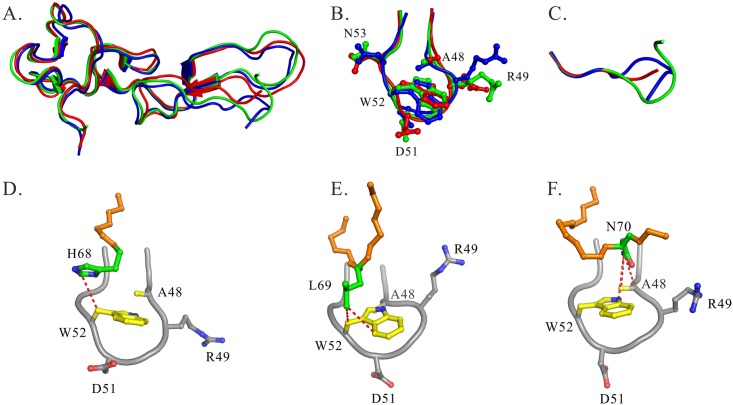
Structural comparisons of ^48^ARGDWN-^65^PRYH, ^48^ARGDWN-^65^PRNGLYG, and ^48^ARGDWN-^65^PRNPWNG mutants. **(A)** Ribbon representation of the averaged structures of ^48^ARGDWN-^65^PRYH, ^48^ARGDWN-^65^PRNGLYG, and ^48^ARGDWN-^65^PRNPWNG mutants are shown in red, blue, and green, respectively. Three two-stranded β sheets (13–14, 20–21, 33–34, 37–38, 43–45, and 55–57) were aligned. The RMSD deviations between ^48^ARGDWN-^65^PRYH and -^65^PRNGLYG mutants and between ^48^ARGDWN-^65^PRYH and -^65^PRNPWNG mutants are 0.681 Å and 0.423 Å. Structural alignments of the ten-residue RGD loop (residues 46 to 54) **(B)** and the C-terminal regions starting from the residue 64 to the C-terminal residue **(C)** of ARGDWN mutants are shown. The interactions between ARGDWN loop and their C-terminal regions of ARGDWN mutants are shown. The interactions of the W52 sidechain (yellow) with the H68 residue of ^48^ARGDWN-^65^PRYH mutant **(D)**, with the L69 residue of ^48^ARGDWN-^65^PRNGLYG mutant **(E)**, and with the N70 residue of ^48^ARGDWN-^65^PRNPWNG mutant **(F)** are shown. Interactions < 4Å are connected with the red broken lines. The sidechains of these C-terminal residues are colored in green.

Superimposing 3D structures of these mutants demonstrated that their overall structures were similar, except for the C-terminal regions and their interactions with the ^48^ARGDWN loop (Fig 4). The structural analysis also indicated that their ^48^ARGDWN loop exhibited similar conformations (Fig 4B). The C-terminal regions of these mutants exhibited distinct conformations: the YH residues of the ^48^ARGDWN-^65^PRYH mutant had an extended structure; the NGLYG residues of the ^48^ARGDWN-^65^PRNGLYG mutant had a turn-like structure; and the NPWN residues of the ^48^ARGDWN-^65^PRNPWNG mutant formed a β-turn structure (Fig 4C). The interactions between the ARGDWN loop and C-terminal regions were extremely different. Our analysis demonstrated that the W52 sidechain of the ^48^ARGDWN-^65^PRYH, ^48^ARGDWN-^65^PRNGLYG, and ^48^ARGDWN-^65^PRNPWNG mutants mainly interacted with H68 of -^65^PRYH (Fig 4D), with L69 of -^65^PRNGLYG (Fig 4E), and with N70 of -^65^PRNPWNG (Fig 4F), respectively. In addition, the A48 sidechain of the ^48^ARGDWN-^65^PRNPWNG mutant interacted with the N70 residue of the C-terminal region, and this interaction was not found in two other mutants. These structural differences may be correlated with their functional differences.

### Interaction differences in the docking models of integrin αIIbβ3-rhodostomin ^48^ARGDWN-^65^P, ^48^ARGDWN-^65^PRNGLYG, and ^48^ARGDWN-^65^PRNPWNG mutant complexes

The docking of ^48^ARGDWN-^65^P, ^48^ARGDWN-^65^PRNGLYG, and ^48^ARGDWN-^65^PRNPWNG mutants into integrin αIIbβ3 was used to simulate their interactions with integrin αIIbβ3. The models of these integrin αIIbβ3 complexes were built using the HADDOCK webserver [33]. The distance and hydrogen bond restraints were derived from the X-ray structure of integrin αIIbβ3 complexed with a GRGDSP hexapeptide (PDB code 3ZE2), including eight key interactions between integrin and the R and D residues (Table C in S1 File). Specifically, the R residue formed a salt bridge with the D224, hydrogen bonds with the Y189 and S225 residues, and a cation-π interaction with the F231 of the αIIb subunit. The carboxylate oxygen of the D residue contacted a Mn^2+^ ion and formed hydrogen bonds with the S123 residues of the β3 subunit. The other carboxyl oxygen of the D residue formed hydrogen bonds with the Y122 and N215 residues of the β3 subunit, and the backbone amide of the D residue formed a hydrogen bond with the R216 residue of subunit β3.

**Fig 5:**
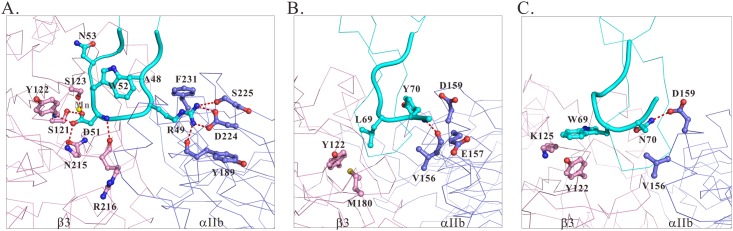
The docking of ^48^ARGDWN-^65^P, ^48^ARGDWN-^65^PRNGLYG, and ^48^ARGDWN-^65^PRNPWNG mutants into integrin αIIbβ3. The contact surface between the ARGDWN loop and integrin αIIbβ3 is shown in **(A)**. The propeller domain of αIIb subunit and the A domain of β3 subunit are shown in purple and pink, respectively. The interacting residues are shown in the ball-and-stick representation, and hydrogen bonds are displayed by broken lines. The C-terminal ^65^PRNGLYG region of ^48^ARGDWN-^65^PRNGLYG **(B)** and the C-terminal ^65^PRNPWNG region of ^48^ARGDWN-^65^PRNPWNG **(C)** mutants are shown.

Using these restraints, we docked Rho ARGDWN mutants to integrin αIIbβ3. The structure cluster was selected based on the lowest Z-score without restraint violations. The Z-score values of the ^48^ARGDWN-^65^P, ^48^ARGDWN-^65^PRNGLYG, and ^48^ARGDWN-^65^PRNPWNG mutants were -1, -1.2, and -1, and their electrostatic energies were -515.9, -569.5, and -630.7 kcal/mol, respectively (Table D in S1 File). This was consistent with the effects of cell adhesion data on integrin αIIbβ3 that ^48^ARGDWN-^65^P and ^48^ARGDWN-^65^PRNPWNG mutants exhibited the lowest and highest inhibitory activities. The resulting structures showed that Rho mutants fitted into a crevice between the propeller domain of the αIIb subunit and the βA domain of the β3 subunit on the αIIbβ3 headpiece. The analysis showed that the docking of these mutants into integrin αIIbβ3 resulted in the same numbers of contacts for the ^48^ARGDWN loop (Table 6). The key contacts included seven hydrogen bonds and two salt bridges between the R and D residues of the RGD motif and integrin. In particular, the contacts of the hydrogen bond and salt bridge between the R49 residue of Rho mutants and the Y189 and D224 residues of the αIIb subunit, and the hydrogen bond between the D51 residue of Rho mutants and the Y122, S123, N215, and R216 residues of the β3 subunit were exhibited by all the mutants (Fig 5A). The major differences between the mutants were the interactions between integrin αIIbβ3 and the C-terminal regions of the Rho mutants (Table 6). The C-terminal region of the ^48^ARGDWN-^65^P deletion mutant did not exhibit any interaction with integrin αIIbβ3 (Table 6). In contrast, the C-terminal regions of the ^48^ARGDWN-^65^PRNGLYG and ^48^ARGDWN-^65^PRNPWNG mutants extensively interacted with integrin αIIbβ3 (Table 6). For example, the C-terminal region of the G71 residue of the ^48^ARGDWN-^65^PRNGLYG mutant formed a hydrogen bond with the V156 residue of the αIIb subunit (Fig 5B and Table 6). The W69 and N70 residues of ^48^ARGDWN-^65^PRNPWNG exhibited cation-π interaction with the K125 residue of the β3 subunit and a hydrogen bond with the D159 residue of the αIIb subunit (Fig 5C and Table 6). In contrast to ^48^ARGDWN-^65^P mutant, the R66 residue of ^48^ARGDWN-^65^PRNGLYG and ^48^ARGDWN-^65^PRNPWNG mutants interacted with the D159 residue of the αIIb subunit. These results suggested that the contents of the C-terminal regions in disintegrins are critical to their abilities to bind to integrin αIIbβ3.

**Table 6:** Summary of the interactions between Rho ^48^ARGDWN-^65^P, ^48^ARGDWN-^65^PRNGLYG, and ^48^ARGDWN-^65^PRNPWNG mutants and integrin αIIbβ3.

Rho Mutant	Integrin		Rho Mutant	Integrin		Rho Mutant	Integrin	
^65^P	αIIb	β3	^65^PRNGLYG	αIIb	β3	^65^PRNPWNG	αIIb	β3
**A48**	F160		**A48**		A218	**A48**	F231	
**R49**	F160	A218	**R49**	Y189^HB^	A218^HB^	**R49**	Y189^HB^	A218
	Y189^HB^			Y190			Y190	
							L192	
	Y190			L192			D224^SB^	
	L192			D224^SB^			S225	
	D224^SB^			S225				
	S225						F231^CP^	
	F231^CP^			F231^CP^				
**G50**	Y190	R216	**G50**	Y190	R216	**G50**	Y190	R216
								D217
		A218			D217			A218
					A218			
**D51**		S121	**D51**		S121	**D51**		S121
					Y122^HB^			
		Y122^HB^			S123^HB^			Y122^HB^
		S123^HB^			R214			S123^HB^
		N215^HB^			N215^HB^			N215^HB^
		R216^HB^			R216^HB^			R216^HB^
		D217			D217^HB^			D217^HB^
		A218			A218			A218
		E220			E220			E220
		Mn^2+^			Mn^2+^			Mn^2+^
**W52**	F160	Y122	**W52**	F160	Y122	**W52**	F160	S123
	Y190	S123		Y190	S123		Y190	
		R214						
**N53**		Y122	**N53**		D126^HB^	**N53**		S123^HB^
		S123^HB^						D126^HB^
		K125						D251
		D126^HB^						
			**R66**	D159		**R66**	D159	
			**L69**		Y122	**W69**	V156	Y122
					M180			K125^CP^
			**Y70**	V156		**N70**	D159^HB^	
				D159				
			**G71**	V156^HB^		**G71**	D159	
				E157				

^HB^, hydrogen bond; ^SB^, salt bridge; ^CP^, cation-π

## Discussion

Many studies have shown that alternations in the RGD loop and C-terminal region of disintegrins affect their binding specificities and affinities [8–19]. In this study, we find that the sequence contents of the RGD loop and C-terminus of disintegrins mutually affected their conformations, resulting in functional and structural differences in integrin binding. We demonstrated that Rho mutants containing a ^48^ARGDWN-^65^PRNPWNG sequence exhibited the highest selectivity in inhibiting integrin αIIbβ3-mediated cell adhesion. Cell adhesion analysis also indicated that the C-terminal region of Rho was highly sensitive to integrin αIIbβ3. Based on the results of cell adhesion, platelet aggregation and the binding of fibrinogen to platelet inhibited by ARGDWN mutants, integrin αIIbβ3 of platelets bound differently to immobilized and soluble fibrinogen. The results of platelet aggregation integrin and αIIbβ3-mediated cell adhesion showed that the R66 and Y67 residues may play important roles in inhibiting the binding of platelet to soluble fibrinogen and the adhesion of integrin αIIbβ3 to immobilized fibrinogen, respectively. NMR structural analysis of ^48^ARGDWN-^65^PRYH, ^48^ARGDWN-^65^PRNGLYG, and ^48^ARGDWN-^65^PRNPWNG mutants showed that their C-terminal regions exhibited distinct conformations. Molecular docking results suggest that the sequence contents and the length of the C-terminal regions in disintegrins are critical to their ability to bind to integrin αIIbβ3. We provide the first structural evidence that the diverse RGD loop and C-terminus of medium disintegrins interact to regulate their conformations, resulting in functional differences in integrin binding.

**Fig 6:**
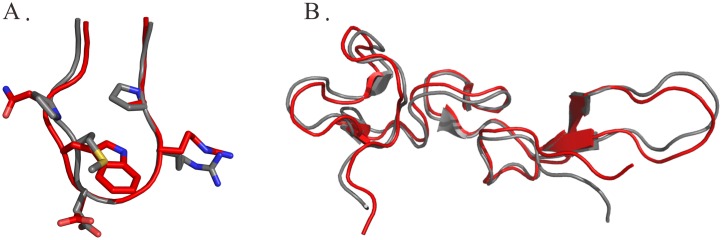
Structural comparison of rho and its ^48^ARGDWN-^65^PRYH mutant. Ribbon representation of the ten-residue RGD loop (residues 46 to 54) **(A)** and all backbone **(B)** of the averaged structures of rho and its ^48^ARGDWN-^65^PRYH mutant are superimposed and shown in grey and red, respectively. The RMS deviation of the secondary structure backbone atom was 0.692 Å. The distances between Cα-to-Cα of the residues 52 and 68 of Rho and its ^48^ARGDWN-^65^PRYH mutant are 12.2 and 8.0 Å, respectively.

The structural analysis of wild-type Rho and its ^48^ARGDWN mutants also showed that a conformational difference existed in the 3D conformation of the RGD loop (Fig 6A). Many studies have demonstrated that a key feature of integrin αIIbβ3 antagonists is the presence of an anionic carboxy-terminal (CO_2_^-^) separated by a spatial chemical moiety and a certain distance from the cationic basic amino-terminal of benzamidine, piperidine, and guanidine [36]. The distance between the anionic (D) and cationic (R) terminals is crucial to the optimal binding affinity and specificity for various integrins. Specifically, the distances between the R and D residues of RGD-containing peptides can be optimally designed for the selective recognition of integrins αIIbβ3, αvβ3, and α5β1 [37]. Therefore, we analyzed the distances between Cα-to-Cα, Cβ-to-Cβ, and Cζ-to-Cγ of the R(i) and D(i+2) residues, and between Cα-to-Cα of the R(i) and X(i+3) residues of Rho, its mutants, and RGD-containing peptides (Table 7). We found that the distance between the Cα-to-Cα of the R(i) and X(i+3) residues was correlated with their integrin specificity. The Cα-to-Cα distances of the R(i) and X(i+3) residues in eptifibatide, an integrin αIIbβ3 antagonist, and in cilengitide, an integrin αvβ3/αvβ5 antagonist, were 7.6 and 5.4 Å, respectively. The average Cα-to-Cα distances of the R(i) and W/M(i+3) residues in Rho ^48^ARGDWN mutants and Rho with a ^48^PRGDMP sequence were 7.3 to 7.6 Å and 6.5 Å, respectively. These results indicated that the Cα-to-Cα distances of the R(i) and X(i+3) residues of the integrin αIIbβ3-specific antagonist were larger than that of the integrin αvβ3 antagonist. This demonstrated that the W52 residue increased the Cα-to-Cα distance between R(i) and W(i+3) of the ^48^ARGDWN motif, resulting in its selectivity to integrin αIIbβ3. Our results were consistent with the previous hypothesis that integrin αIIbβ3-specific disintegrin prefers a larger Cα(i)-to-Cα(i+3) distance in its RGDX motif [8].

**Table 7:** Comparison of the C_α_(R_i_)-C_α_(D_i+2_), C_β_(R_i_)-C_β_(D_i+2_), C_ζ_(R_i_)-C_γ_(D_i+2_), and C_α_(R_i_)-C_α_(X_i+3_) distances (Å) of integrin ligands.

Ligands	RGD motif	C_α_(R_i_)–C_α_(D_i+2_)	C_β_(R_i_)–C_β_(D_i+2_)	C_ζ_(R_i_)–C_γ_(D_i+2_)	C_α_(R_i_)–C_α_(X_i+3_)
Eptifibatide^a^	HrgGDWP	6.9	9.2	15.5	7.6
RGD peptide^b^	GRGDSP	7.1	9.4	15.5	8.0
Cilengitide^c^	c(-RGDf[NMe]V-)	6.4	8.9	13.7	5.4
^48^ARGDWN-^65^PRYH	ARGDWN	6.5±0.4	8.6±0.6	12.7±1.1	7.3±0.5
^48^ARGDWN-^65^PRNGLYG	ARGDWN	6.5±0.3	8.9±0.3	13.0±1.1	7.6±0.3
^48^ARGDWN-^65^PRNPWNG	ARGDWN	6.5±0.3	8.8±0.4	12.4±1.1	7.6±0.4
Rhodostomin^d^	PRGDMP	6.3±0.5	8.1±1.0	11.9±1.4	6.5±0.6

^a^ The antagonist of integrin αIIbβ3; PDB code: 2VDN

^b^ The antagonist of integrin αIIbβ3; PDB code: 3ZE2

^c^ The antagonist of integrins αvβ3 and αvβ5; PDB code: 1L5G

^d^ PDB code: 2PJF

Many studies have shown that the C-terminal tails of disintegrins are located in the proximity of the RGD loop, the integrin-binding loop, and that the C-terminal regions of disintegrins play synergistic roles in interacting with RGD-binding integrins [16, 18, 20, 21, 25]. For example, C-terminal W67 of flavoridin with an ^48^ARGDFP motif is close to D55 [21], C-terminal Y67 of Rho with a ^48^PRGDMP motif is close to D55 [25], and C-terminal W67 of trimestatin with a ^48^ARGDNP motif is close to P53 [18]. The structural analysis of wild-type Rho and its ^48^ARGDWN mutants also showed that a conformational difference existed in their RGD loop and C-terminal region (Fig 6B). In contrast to that of Rho, structural analyses of the Rho ^48^ARGDWN-^65^PRYH mutant indicated that C-terminal H68 is close to W52. C-terminal L69 of the ^48^ARGDWN-^65^PRNGLYG mutant is close to W52, and the C-terminal N70 residue of the ^48^ARGDWN-^65^PRNGWNG mutant is close to W52 and A48. We also found that the ^67^NPWN region of the ^48^ARGDWN-^65^PRNPWNG mutant formed a type I β-turn, which was not found in other C-terminal mutants. These results suggest that the sequence contents of the C-terminal region and RGD loop of disintegrins are important for their 3D conformation and mutual interactions. These structural differences may be correlated with their functional differences.

Integrins are known for their ability to bind multiple ligands due to flexibility in their binding sites. Many studies showed that integrin αIIbβ3 adhesion on fibrinogen is mediated by recognition sequences RGDF (Aα95–98), RGDS (Aα572–575), and HHLGGAKQAGDV (γ 400–411) of fibrinogen [38–41]. In particular, different recognition sites of soluble and immobilized fibrinogen are used for their binding to integrin αIIbβ3 [38, 39]. For example, integrin αIIbβ3 binds to soluble fibrinogen mainly through HHLGGAKQAGDV (γ 400–411). Integrin αIIbβ3 binds to immobilized fibrinogen through not only HHLGGAKQAGDV (γ 400–411) but also RGDF (Aα95–98). In contrast to integrin αvβ3 adhesion on fibrinogen, it is only mediated by the carboxyl-terminal RGDS site of the Aα chain [4141]. Our findings revealed that Rho ^48^ARGDWN mutants selectively inhibited integrin αIIbβ3 to immobilized and soluble fibrinogen, and the incorporation of C-terminal NPWNG increased its inhibitory activity to immobilized fibrinogen. However, it is likely that the specificity of Rho ARGDWN mutants with C-terminal PRNPWNG sequence towards αIIbβ3 antagonism could be due to better competition with not only RGD but also the γ-chain ligands as well. Although functional and structural differences in ARGDWN mutants and their integrin αIIbβ3 complexes were found from our study, it is uncertain that these interactions may take place in vivo and are affected by the ionic milieu. The effect of recognition by the inside-out signaling on integrin cannot be also excluded as well.

In conclusion, our functional and structural analyses demonstrated that the RGD loop and C-terminus of rhodostomin mutants interact with each other. The amino acid sequences of the RGD loop and C-terminal regions in medium disintegrins are important for their interactions and abilities to the binding of integrin αIIbβ3 to immobilized and soluble fibrinogen. These findings provide new insights into the structure-based drug design of integrin αIIbβ3 antagonist by using the disintegrin scaffold, and they serve as a basis for exploring the structure-function relationships of RGD-binding integrins and their ligands.

## Supporting information

S1 File **Figure A. Mass spectra of recombinant Rho and its mutants**. **(A)** mass spectrum of ^48^PRGDMP-^65^PR mutant, **(B)** mass spectrum of ^48^PRGDMP-^65^PRYH (Rho), **(C)** mass spectrum of ^48^PRGDMP-^65^PRNGLYG mutant, **(D)** mass spectrum of ^48^PRGDMP-^65^PRNPWNG mutant, **(E)** mass spectrum of ^48^ARGDWN-^65^P mutant, **(F)** mass spectrum of ^48^ARGDWN-^65^PR mutant, **(G)** mass spectrum of ^48^ARGDWN-^65^PRY mutant, **(H)** mass spectrum of ^48^ARGDWN-^65^PRYH mutant, **(I)** mass spectrum of ^48^ARGDWN- ^65^PRNGLYG mutant, and **(J)** mass spectrum of ^48^ARGDWN-^65^PRNPWNG mutant.
**Figure B. Summary of NMR data for** ^**48**^**ARGDWN-**^**65**^**PRYH (A)**, ^**48**^**ARGDWN-**^**65**^**PRNGLYG (B), and** ^**48**^**ARGDWN-**^**65**^**PRNPWNG (C) mutants**. The intensities of NOEs are represented by the thickness of the blocks.
**Figure C. Amide strip plots and 2D** ^**1**^**H-**^**1**^**H NOESY spectra of Rho** ^**48**^**ARGDWN mutants. (A)** Amide strip plots of W52 and N67 to G71 of ^48^ARGDWN-^65^PRNPWNG at pH 6.0. The dNN (i, i +1) and dαN (i, i +1) NOE connectivities are shown. 2D ^1^H-^1^H NOESY spectra of ^48^ARGDWN-^67^YH **(B)** in 100% D_2_O, ^48^ARGDWN-^67^NGLYG **(C)** in 100% D_2_O and ^48^ARGDWN-^67^NPWNG **(D, E)** in H_2_O: D_2_O (9:1, v/v) show NOE connections between RGD loop and C-terminal region. **(D)** NOE connections between sidechain NH of W52 and the protons of the RGD loop and C-terminal region. **(E)** NOE connections between the protons of the RGD loop and C-terminal region. The NOEs between the ARGDWN loop and their C-terminal regions were shown in red.
**Table A. Molecular weights of recombinant Rho and its mutants**.
**Table B. Inhibition of platelet aggregation by Rho and its C-terminal mutants**.
**Table C. Summary of the interactions between the GRGDSP peptide and integrin αIIbβ3 (PDB code: 3ZE2)**.
**Table D. Statistical analysis of integrin αIIbβ3–Rho mutants docking results obtained by Haddock webserve**.
(DOC)
