# Supplementary material for: Effects of the RGD loop and C-terminus of rhodostomin on regulating integrin αIIbβ3 recognition
Source: PLoS One. 2017 Apr 11;12(4):e0175321. doi: 10.1371/journal.pone.0175321 (PMC5388508; doi:10.1371/journal.pone.0175321)
Supplement: S1 File — Figure A. Mass spectra of recombinant Rho and its mutants. (A) mass spectrum of 48PRGDMP-65PR mutant, (B) mass spectrum of 48PRGDMP-65PRYH (Rho), (C) mass spectrum of 48PRGDMP-65PRNGLYG mutant, (D) mass spectrum of 48PRGDMP-65PRNPWNG mutant, (E) mass spectrum of 48ARGDWN-65P mutant, (F) mass spectrum of 48ARGDWN-65PR mutant, (G) mass spectrum of 48ARGDWN-65PRY mutant, (H) mass spectrum of 48ARGDWN-65PRYH mutant, (I) mass spectrum of 48ARGDWN- 65PRNGLYG mutant, and (J) mass spectrum of 48ARGDWN-65PRNPWNG mutant. Figure B. Summary of NMR data for 48ARGDWN-65PRYH (A), 48ARGDWN-65PRNGLYG (B), and 48ARGDWN-65PRNPWNG (C) mutants. The intensities of NOEs are represented by the thickness of the blocks. Figure C. Amide strip plots and 2D 1H-1H NOESY spectra of Rho 48ARGDWN mutants. (A) Amide strip plots of W52 and N67 to G71 of 48ARGDWN-65PRNPWNG at pH 6.0. The dNN (i, i +1) and dαN (i, i +1) NOE connectivities are shown. 2D 1H-1H NOESY spectra of 48ARGDWN-67YH (B) in 100% D2O, 48ARGDWN-67NGLYG (C) in 100% D2O and 48ARGDWN-67NPWNG (D, E) in H2O: D2O (9:1, v/v) show NOE connections between RGD loop and C-terminal region. (D) NOE connections between sidechain NH of W52 and the protons of the RGD loop and C-terminal region. (E) NOE connections between the protons of the RGD loop and C-terminal region. The NOEs between the ARGDWN loop and their C-terminal regions were shown in red. Table A. Molecular weights of recombinant Rho and its mutants. Table B. Inhibition of platelet aggregation by Rho and its C-terminal mutants. Table C. Summary of the interactions between the GRGDSP peptide and integrin αIIbβ3 (PDB code: 3ZE2). Table D. Statistical analysis of integrin αIIbβ3–Rho mutants docking results obtained by Haddock webserve. (DOC) [file pone.0175321.s001.doc]

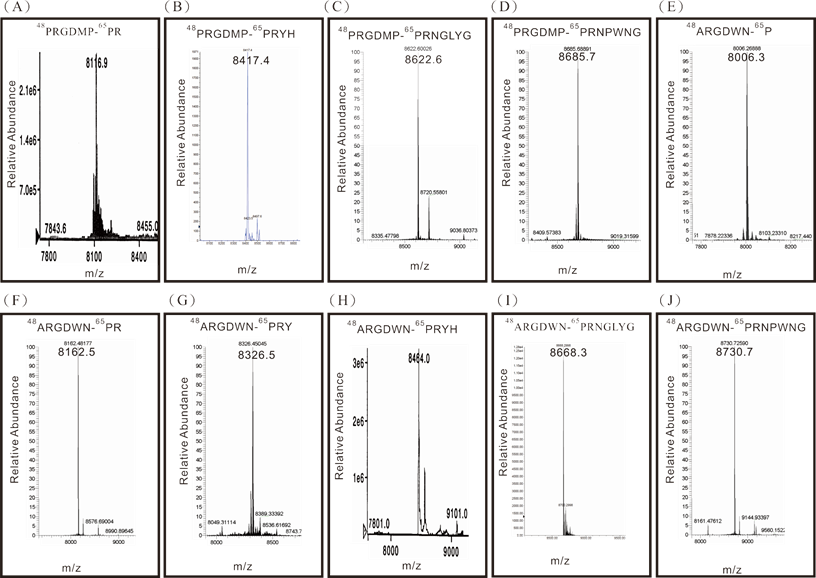


**Figure A. Mass spectra of recombinant Rho and its mutants.**

**(A)** mass spectrum of ^48^PRGDMP-^65^PR mutant, **(B)** mass spectrum of ^48^PRGDMP-^65^PRYH (Rho), **(C)** mass spectrum of ^48^PRGDMP-^65^PRNGLYG mutant, **(D)** mass spectrum of ^48^PRGDMP-^65^PRNPWNG mutant, **(E)** mass spectrum of ^48^ARGDWN-^65^P mutant, **(F)** mass spectrum of ^48^ARGDWN-^65^PR mutant, **(G)** mass spectrum of ^48^ARGDWN-^65^PRY mutant, **(H)** mass spectrum of ^48^ARGDWN-^65^PRYH mutant, **(I)** mass spectrum of ^48^ARGDWN- ^65^PRNGLYG mutant, and **(J)** mass spectrum of ^48^ARGDWN-^65^PRNPWNG mutant.

**
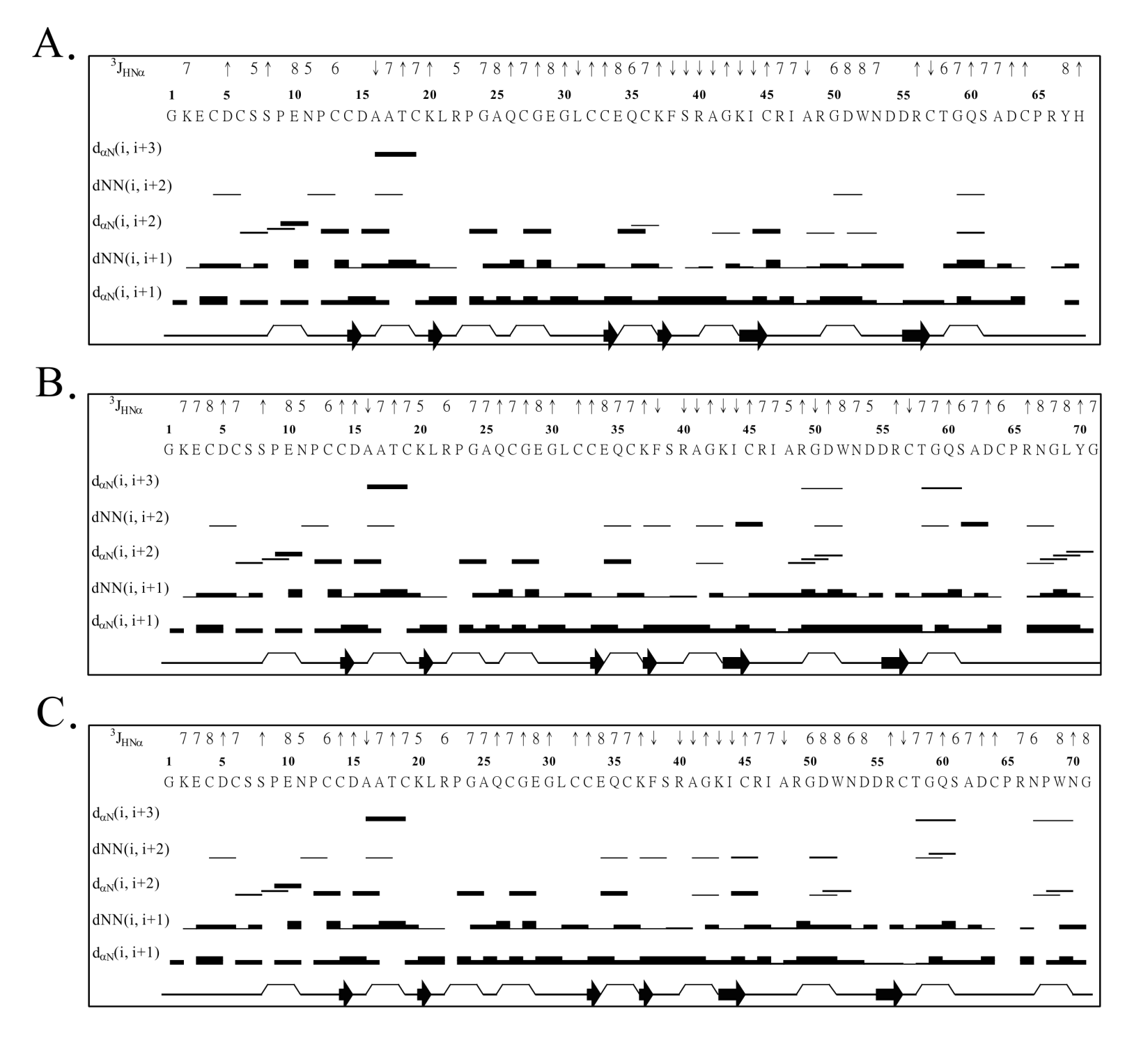
**

**Figure B. Summary of NMR data for ^48^ARGDWN-^65^PRYH (A), ^48^ARGDWN-^65^PRNGLYG (B), and ^48^ARGDWN-^65^PRNPWNG (C) mutants.**

The intensities of NOEs are represented by the thickness of the blocks.


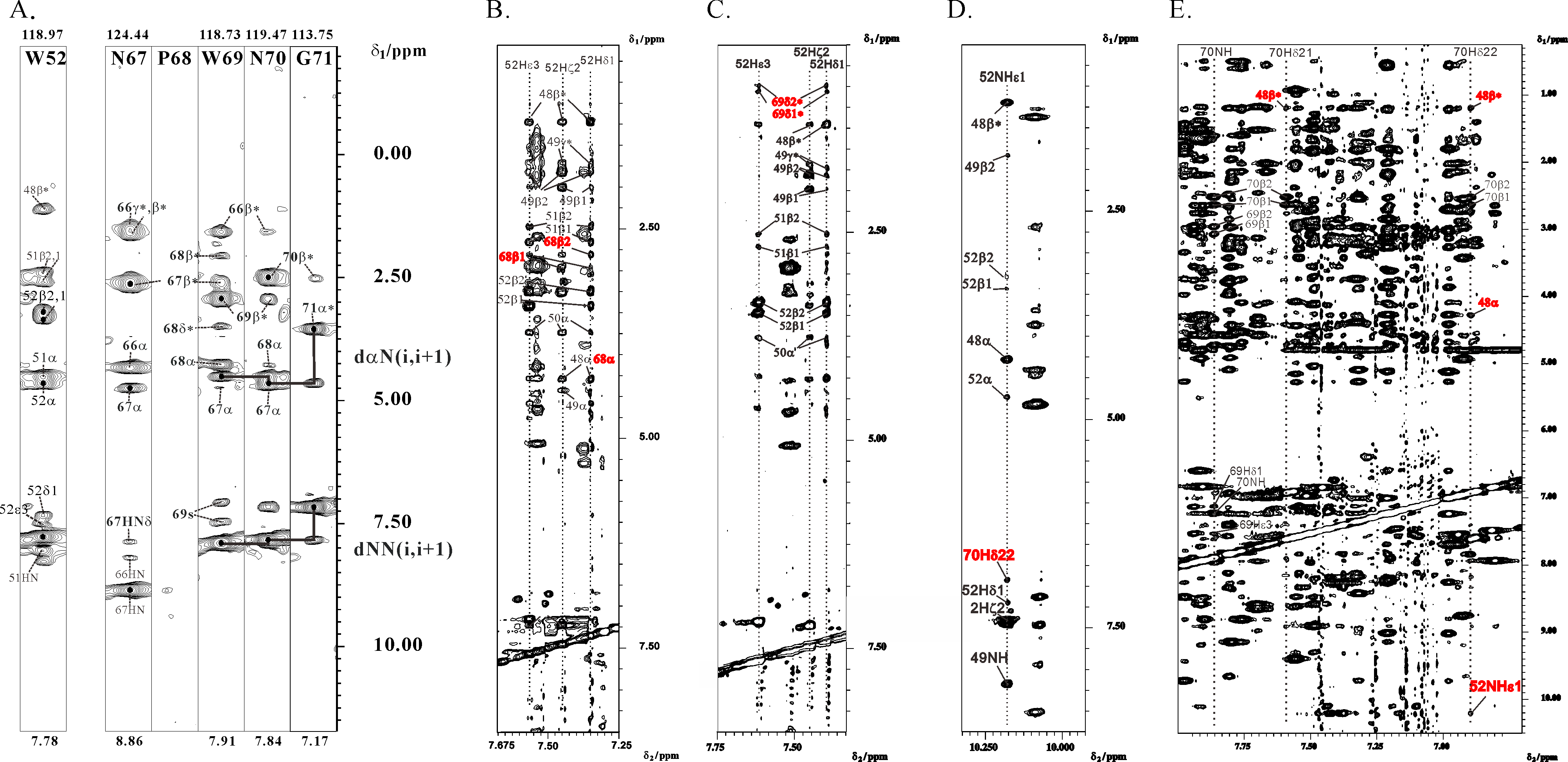


**Figure C. Amide strip plots and 2D ^1^H-^1^H NOESY spectra of Rho ^48^ARGDWN mutants.**

**(A)** Amide strip plots of W52 and N67 to G71 of ^48^ARGDWN-^65^PRNPWNG at pH 6.0. The dNN (i, i +1) and dαN (i, i +1) NOE connectivities are shown.

2D ^1^H-^1^H NOESY spectra of ^48^ARGDWN-^67^YH **(B)** in 100% D_2_O, ^48^ARGDWN-^67^NGLYG **(C)** in 100% D_2_O and ^48^ARGDWN-^67^NPWNG **(D, E)** in H_2_O: D_2_O (9:1, v/v) show NOE connections between RGD loop and C-terminal region. **(D)** NOE connections between sidechain NH of W52 and the protons of the RGD loop and C-terminal region. **(E)** NOE connections between the protons of the RGD loop and C-terminal region. The NOEs between the ARGDWN loop and their C-terminal regions were shown in red.

**Table A. Molecular weights of recombinant Rho and its mutants.**

| Proteins | Sequence of Rho Mutants | | Molecular Weight | | |
| --- | --- | --- | --- | --- | --- |
|  | RGD Loop | C-terminus | Calculated | Experimental | Deviation |
| ^48^PRGDMP-^65^PR | PRGDMP | PR | 8117.1 | 8116.9 | -0.2 |
| ^48^PRGDMP-^65^PRYH | PRGDMP | PRYH | 8417.4 | 8417.4 | +0.0 |
| ^48^PRGDMP-^65^PRNGLYG | PRGDMP | PRNGLYG | 8621.7 | 8622.6 | +0.9 |
| ^48^PRGDMP-^65^PRNPWNG | PRGDMP | PRNPWNG | 8685.7 | 8685.7 | +0.0 |
| ^48^ARGDWN-^65^P | ARGDWN | P | 8006.9 | 8006.3 | -0.6 |
| ^48^ARGDWN-^65^PR | ARGDWN | PR | 8163.0 | 8162.5 | -0.5 |
| ^48^ARGDWN-^65^PRY | ARGDWN | PRY | 8326.2 | 8326.5 | +0.3 |
| ^48^ARGDWN-^65^PRYH | ARGDWN | PRYH | 8463.4 | 8464.0 | +0.6 |
| ^48^ARGDWN-^65^PRNGLYG | ARGDWN | PRNGLYG | 8667.6 | 8668.3 | +0.7 |
| ^48^ARGDWN-^65^PRNPWNG | ARGDWN | PRNPWNG | 8731.6 | 8730.7 | -0.9 |

**Table B. Inhibition of platelet aggregation by Rho and its C-terminal mutants**

|  |  | Platelet aggregation | | |
| --- | --- | --- | --- | --- |
| RGD Loop | C-terminus | IC_50_(nM) | | *Q* |
| ^48^PRGDMP | ^65^PRYH | 83.2 | ±10.4 | 1.0 |
| ^48^PRGDMP | ^65^PR | 155.2 | ±6.2 | 1.9 |
| ^48^PRGDMP | ^65^PRNGLYG | 96.9 | ±10.3 | 1.2 |
| ^48^PRGDMP | ^65^PRNPWNG | 130.9 | ±25.2 | 1.6 |
| Folds | |  |  | 1.0-1.9 |

*Q ratio* = IC_50_ [Rho mutants] / IC_50_ [Rho]

**Table C. Summary of the interactions between the GRGDSP peptide and integrin αIIbβ3 (PDB code: 3ZE2).**

| GRGDSP | αIIb subunit | β3 subunit |
| --- | --- | --- |
| R | Y189^HB^, Y190, L192, D224^SB^, S225^HB^, F231^CP^ | A218 |
| G | Y190 | R216, A218 |
| D |  | S121, Y122^HB^, S123^HB^, R214, N215^HB^, R216^HB^, D217, A218, E220, Mn^2+^ |
| S |  | S123 |
| P |  | Y122, S123, D126 |

^HB^, hydrogen bond; ^SB^, salt bridge; ^CP^, cation-π

**Table D. Statistical analysis of integrin αIIbβ3–Rho mutants docking results obtained by Haddock webserve**

|  | Proteins | | | | | |
| --- | --- | --- | --- | --- | --- | --- |
|  | ^48^ARGDWN-  ^65^P^a^ | | ^48^ARGDWN-  ^65^PRNGLYG^b^ | | ^48^ARGDWN-  ^65^PRNPWNG^c^ | |
| Electrostatic energy (kcal/mol) | -515.9 | ±21.4 | -569.5 | ±28.0 | -630.7 | ±21.7 |
| Van der Waals energy (kcal/mol) | -40.6 | ±4.6 | -54.1 | ±3.5 | -41.9 | ±4.4 |
| Restraints violation energy (kcal/mol) | 1.7 | ±0.1 | 2.4 | ±0.3 | 4.0 | ±3.8 |
| HADDOCK score | -149.0 | ±5.3 | -177. | ±6.8 | -176.1 | ±16.1 |
| Z-Score | -1.0 | | -1.2 | | -1.0 | |
| Cluster size | 178 | | 94 | | 44 | |
| Buried Surface Area (Å^2^) | 1314.8 | ±23.2 | 1919.7 | ±39.3 | 1849.3 | ±64.2 |
| RMSD | 0.6 | ±0.4 | 0.4 | ±0.2 | 0.5 | ±0.3 |

^a^ A C-terminal deletion model of the ^48^ARGDWN-^65^P mutant was constructed from the average structure of rho ^48^ARGDWN-^65^PRYH mutant in PyMOL.

^b^, ^c^ 20 NMR structures were used to dock.
